# Complete mitochondrial genome of *Ciconia nigra* (Ciconiiformes: Ciconiidae), a threatened stork in China

**DOI:** 10.1080/23802359.2019.1640082

**Published:** 2019-07-13

**Authors:** Wu-Long Liang, Jun Zhu, Zhu-Mei Ren

**Affiliations:** a School of Life Science, Shanxi University, Taiyuan, China;; b Biodiversity Research Center of Shanxi, Taiyuan, China

**Keywords:** *Ciconia nigra*, Ciconiidae, mitochondrial genome, phylogeny

## Abstract

The complete mitochondrial genome (mitogenome) of black stork *Ciconia nigra* from North China was sequenced by shotgun genome-skimming method. The mitogenome of *C. nigra* was 17,787 bp in length and consists of 13 protein-coding genes, 22 tRNAs, two rRNAs, and one non-coding control region (D-loop). All protein-coding genes initiate with ATG codon except for *ND2*, *ND3, and COX1*, which uses ATA, ATC, and GTG as their initiation codons, respectively. The termination codon of protein-coding genes shows rich diversity with six termination codons (TAA, AGG, AGA, TAG, T, and A). The phylogenetic trees based on 13 protein-coding genes showed that *Ciconia* formed a monophyletic group, which was sister to the clade clustered by Threskiorothidae species.

*Ciconia nigra* (Aves: Ciconiiformes: Ciconiidae), also known as black stork, is one of the largest waders and widely distributed in the Palearctic region (Chevallier et al. 2011). This species always makes a long-distance migration between Europe and Africa or West Asia and India (Hancock et al. 2010), except that they partially resided in Spain and South Africa (Santiago et al. 2006). However, the populations of *C. nigra* are declining in parts of their range (Konovalov et al. 2015) and has been considered as Least Concern species in IUCN Red List of Threatened Species ver 3.1 (2017). So far, only two complete mitochondrial genomes (mitogenomes) of *C. nigra* from South Korea and South China were reported (Liu et al. 2016; Lee et al. 2017). Here, the complete mitogenome of *C. nigra* from North China was sequenced and the relationship with other Ciconiiformes species was constructed.

The feather was collected from a healthy adult individual of *Ciconia nigra* in Taiyuan Zoo, Shanxi, China. This individual is originally from Wutai County and the feather and total DNA were deposited at School of Life Science, Shanxi University (Voucher No. Ren_Z1). The mitogenome sequence of *C. nigra* was obtained with the shotgun genome-skimming method on an Illumina HiSeq4000 platform (Zimmer and Wen 2015) and annotated referencing the complete mitogenomes of *C. nigra*, *C. boyciana*, and *C. ciconia* from GenBank.

The complete mitogenome of *Ciconia nigra* (Accession no. MK818509) is a double-stranded circular DNA with 17,787 bp in length. The A + T content (54.3%) is a little higher than G + C content (45.7%), which is basically consistent with those of other Ciconiidae species (Liu et al. 2016; Lee et al. 2017). The mitogenome of *C. nigra* comprised 13 protein-coding genes (*ND1-6* and *ND4L*, *COX1-3*, *ATP6* and *ATP8*, and *Cytb*), 22 tRNAs, two rRNAs (*12S* and *16S* rRNA), and one noncoding region (*D-*loop). Nine of the 13 protein-coding genes have a typical ATG initiation codon except for *ND2*, *ND3* and *COX1* with ATA, ATC, and GTG as their initiation codons, respectively. Among these genes, *ND4L*, *ND6*, *ATP6,* and *ATP8* terminated with the codon TAA and *ND1* and *COX1* with AGG as their stop codon. However, *ND5* and *Cytb* used AGA and TAG as their termination codon, respectively. However, other four genes (*ND2*, *ND4*, *COX2,* and *COX3*) terminated with a single T and *ND3* ended with a single A.

Based on the 13 protein-coding genes, we used the maximum-likelihood method to construct the phylogenetic trees of the Ciconiiformes species under the GTRGAMMA model and with 1000 bootstrap replicates using two Anseriformes species as outgroups (Figure 1). The phylogenetic analysis well supported the monophyly of the three families of the order Ciconiiformes and the Genus *Ciconia* with high bootstrap values. *Ciconia boyciana* and *C. ciconia* were sister species and the three individuals of *C. nigra* closely grouped into a clade with very tiny variation. The genetic variation of *C. nigra* population might be further examined to choose more samples and data in the future.

**Figure 1.**
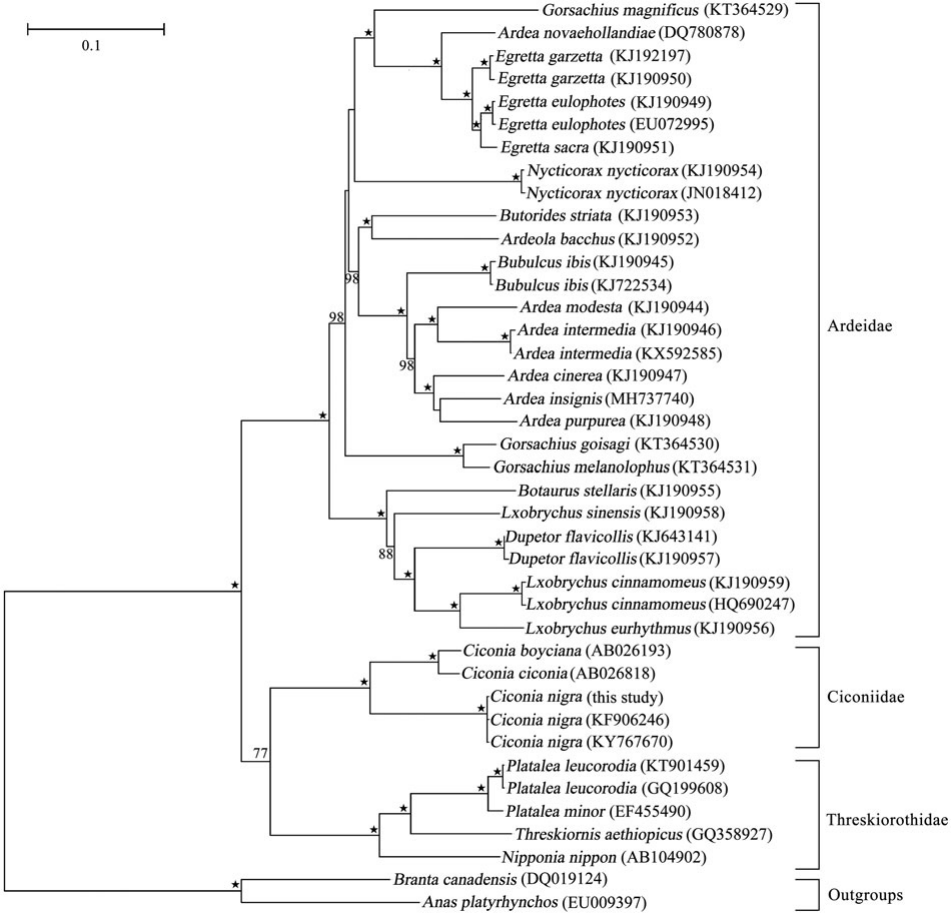
Maximum likelihood phylogenetic tree of *Ciconia nigra* and other representative Ciconiiformes species based on 13 protein-coding genes. Numbers associated with branches are BS > 75, and “★” represents nodes with 100% BS.
